# Optimizing Nutritional Strategies to Prevent Necrotizing Enterocolitis and Growth Failure after Bowel Resection

**DOI:** 10.3390/nu13020340

**Published:** 2021-01-24

**Authors:** Laura Moschino, Miriam Duci, Francesco Fascetti Leon, Luca Bonadies, Elena Priante, Eugenio Baraldi, Giovanna Verlato

**Affiliations:** 1 Neonatal Intensive Care Unit, Department of Women’s and Children’s Health, University Hospital of Padova, 35128 Padova, Italy; lauramoschino13@gmail.com (L.M.); luca.bonadies@aopd.veneto.it (L.B.); elena.priante@aopd.veneto.it (E.P.); eugenio.baraldi@unipd.it (E.B.); 2 Pediatric Surgery Unit, Department of Women’s and Children’s Health, University Hospital of Padova, 35128 Padova, Italy; ducimiriam@gmail.com (M.D.); francesco.fascettileon@unipd.it (F.F.L.)

**Keywords:** necrotizing enterocolitis, short bowel syndrome, human milk, nutrition, surgical management, bowel sparing

## Abstract

Necrotizing enterocolitis (NEC), the first cause of short bowel syndrome (SBS) in the neonate, is a serious neonatal gastrointestinal disease with an incidence of up to 11% in preterm newborns less than 1500 g of birth weight. The rate of severe NEC requiring surgery remains high, and it is estimated between 20–50%. Newborns who develop SBS need prolonged parenteral nutrition (PN), experience nutrient deficiency, failure to thrive and are at risk of neurodevelopmental impairment. Prevention of NEC is therefore mandatory to avoid SBS and its associated morbidities. In this regard, nutritional practices seem to play a key role in early life. Individualized medical and surgical therapies, as well as intestinal rehabilitation programs, are fundamental in the achievement of enteral autonomy in infants with acquired SBS. In this descriptive review, we describe the most recent evidence on nutritional practices to prevent NEC, the available tools to early detect it, the surgical management to limit bowel resection and the best nutrition to sustain growth and intestinal function.

## 1. Background

Short bowel syndrome (SBS) is a state of malabsorption defined as the need for parenteral nutrition (PN) for >60 days after bowel resection or as a bowel length of less than 25% of expected [1]. SBS is the principal cause of intestinal failure (IF) in the pediatric age [2]. The incidence of SBS has been estimated to be 24.5/100.000 births per year [3], but it may reach 7/1000 births in preterm newborns with birth weight (BW) <1500 g and [4] even a higher rate (22.1/1000 births) considering the neonatal intensive care units (NICUs) with a much greater prevalence of premature infants (353.7/100,000 live births) [5]. However, its real incidence and prevalence are very difficult to determine due to the rarity of the condition and differences in definitions used.

SBS may result from massive resection of the small intestine [6] with necrotizing enterocolitis (NEC) as the leading cause in the neonatal age [7]. The overall incidence of NEC is 1 per 1000 live births [8] but it varies between centers and reaches 11% in very low birth weight infants (VLBWI) [9] and 22% in extremely preterm infants <1000 g [10]. Despite improvements in neonatal care in recent decades, the incidence of NEC and of NEC requiring surgery seems relatively unchanged [11,12]. Indeed, rates of preterm birth (gestational age GA < 37 weeks) have increased globally [13] with a consequent higher risk of developing prematurity-related morbidities such as NEC itself. NEC is a multifactorial acquired devastating intestinal disease that occurs due to bowel immaturity mainly in enterally fed preterm newborns and due to excessive inflammatory responses. It can have a mild presentation (abdominal distention only) or a typical association of signs and symptoms (emesis, bloody stools, intestinal pneumatosis, abdominal tenderness) as described according to Bell’s staging and its modification by Walsh et al. [14,15]. Ultimately, NEC can progress to full intestinal necrosis [16] with a high associated mortality (around 50% in those born extremely preterm) [17] and several comorbidities in those who survive, with SBS developing in 42% of those requiring surgery [18].

Due to the lack of a specific therapy against NEC-related bowel damage, rationalized, targeted, and prolonged prevention programs and early recognition are fundamental to limit bowel loss. In fact, the length of the residual bowel, as well as the presence of the ileo-caecal valve and of the colon are the main factors influencing the chances of weaning from PN [19,20,21,22].

In this descriptive review, we report the most recent evidence to prevent and early detect NEC, the surgical management to limit bowel resection and SBS development, and the best nutrition to sustain growth and enhance intestinal function.

## 2. Factors to Prevent NEC Development

There are several measures which were demonstrated to be promising in reducing the incidence of NEC in premature infants, although with variable evidence.

### 2.1. Prenatal and Perinatal Factors

Starting from the womb, clinical maternal chorioamnionitis seems to be significantly associated with NEC, while this does not appear to be true for histological chorioamnionitis without fetal involvement (funisitis, fetal surface vessel angiitis, increased inflammatory markers in umbilical cord or fetal blood). Despite the data are still preliminary, there is a good available evidence that supports a role of antenatal inflammation in NEC pathophysiology [23,24]. It is possible that as in bronchopulmonary dysplasia (BPD), maternal chorioamnionitis plays a different role in NEC pathophysiology depending on its onset (acute or chronic), its association with severe inflammatory response syndrome (SIRS) of the fetus, and the involved pathogen. Recently, Ureaplasma species have been acknowledged as major causative pathogens of both BPD and NEC, most likely by inducing pro-inflammatory factors and down-regulating the immune system [25].

These data could partially explain why antenatal corticosteroids appear to be effective in NEC prevention. From randomized clinical trials (RCT), a decreased risk of NEC is seen with antenatal corticosteroids in pregnant women at risk of preterm birth [26]. A recent review and meta-analysis of nine observational studies, however, demonstrated that antenatal corticosteroid use before 25 weeks’ gestation (which is controversial), does not influence the rate of NEC ≥stage II of Bell [27].

Mode of delivery is one of the first determinants of gut microbiota, together with gestational age, antibiotic treatment, and diet [28]. Compared to infants born vaginally, those born via cesarean section show decreased intestinal population of *Bifidobacteria* and *Bacteroides* and increased population of *Clostridium difficile* [29]. However, in a secondary analysis of data from a randomized controlled trial, mode of delivery was not significantly associated with development of NEC in neonates of women who were at imminent risk of delivery at <32 gestational weeks (GW) [30,31]. Indeed, despite fecal bacterial microflora differs significantly depending on the delivery route, the more significant change in colonization seems to occur at a later stage, typically after 2–6 weeks of age, at the time of NEC onset [32]. This is confirmed by the evidence that NEC does not arise in utero despite the presence of microbes in meconium, but it necessitates of other factors determining a certain level of dysbiosis to develop [33,34]. At the moment, there is not enough evidence to suggest a mode of delivery is better than the other to prevent the development of NEC.

As regards delayed cord clamping (DCC), this method was found to reduce the incidence of NEC in a Cochrane review [35]. However, the effects of DCC on prevention of NEC are not fully understood and warrant further investigation.

Finally, a lower birth weight at delivery increases the risk of NEC, with placental disease predisposing the severely growth-restricted neonate to the disease [36]. Additionally, in antenatally identified pregnancies at risk of fetal growth restriction, abnormal Doppler velocimetry in the umbilical artery (absent/reverse end-diastolic flow) is a useful guide to predict NEC and mortality in the early neonatal period [37,38].

### 2.2. Post-Natal Factors

When it comes to post-natal life, other protective factors have come into focus, and the importance of an optimized nutrition has been highlighted.

#### 2.2.1. Feeding Management

Starting from the feeding type, since the 1990s, human milk (maternal or donor) has proven to lower the risk of NEC compared with bovine protein-based formula [39]. Maternal breast milk is recommended for preterm and low birth weight infants as it has been demonstrated to attenuate the toll-like receptor 4 mediated pro-inflammatory response, typical hallmark in NEC pathogenesis, by activating the receptor for epidermal growth factor (EGFR) and thus resulting in enhanced mucosal healing, intestinal stem cell proliferation and decreased enterocyte apoptosis [28,40]. In the case of insufficient supply, maternal breast milk can be replaced by donor human milk, despite pasteurization and freezing of the latter reduce some of the protective benefits of the former [41,42]. The incidence of NEC, indeed, has been described as 6–10 times higher in exclusively formula-fed infants compared to the exclusively breastfed ones [43,44,45]. Human breast milk, which has an osmolarity of around 300 mOsm/L, acts by increasing proteolytic enzymes and decreasing gastric pH, thus determining less pathogenic bacterial flora and improving epithelial membrane and tight junctions. In addition, in preterm infants it stimulates peristalsis and gut motility, together with the immune system through secretary IgA, lactoferrin, growth hormones and oligosaccharides, thereby lowering the extent of microbial dysbiosis [33]. By contrast, preterm infant formula appears to alter the intestinal flora selecting potential pathogenic bacteria such as Clostridia and Proteobacteria [46], despite the relatively safe osmolarity of most products (from 210 up to 270 mOsm/L) [47]. Interestingly, the positive effects of maternal milk appear to be dose-dependent, with higher intake of human milk leading to higher protection from NEC [45,48].

Multi-nutrient fortification adds protein, vitamins, and other minerals to human milk, therefore preventing nutrient deficits and extra-uterine growth restriction in exclusively breast milk-fed preterm infants [49,50]. A Cochrane review published in 2016 concluded that there is only low-quality evidence that multi-nutrient fortified breast milk compared with unfortified breast milk does not increase the risk of NEC (RR 1.57, 95% CI 0.76 to 3.23; 11 studies, 882 infants) [51]. Similar findings have emerged from a recent RCT in South India, where standard fortification of pasteurized donor human milk did not increase the incidence of NEC compared to the unfortified one [52]. Commonly, multi-nutrient fortifiers to breast milk derive from bovine milk, but fortification of breast milk feeds with human milk-derived fortifier is available. Nevertheless, a Cochrane review of one randomized trial showed that the latter does not seem to decrease the risk of NEC, feeding intolerance, late-onset sepsis or death, compared to bovine milk-derived fortifier [53,54].

In recent years two new fortification strategies have gained popularity to optimize macronutrient intake, improve growth and minimize feeding intolerance and NEC [55,56]. The first is adjustable fortification based on blood urea nitrogen levels to adjust fortifier strength. The second is target and customized fortification through human milk analyzers that fortifies macronutrients individually to achieve the desired intake [57]. Composition of native breast milk, indeed, has individual inter- and intra-sample variation. Targeting components of fortification ensures that current osmolarity recommendations are followed, as fortification could increase the osmolarity of breast milk [58]. The addition of 1 g of carbohydrates (glucose polymer), 1 g of hydrolyzed protein, or 1 g of whey protein per 100 mL breast milk, seem to determine an average increase in osmolality of 20, 38, and 4 mOsm/kg respectively. Recently, prediction models to estimate osmolality values after fortification have been published [59,60].

Oral colostrum, both of bovine or maternal origin, is rich in nutrients and bioactive factors. Although intact bovine colostrum added to donor human milk appeared superior to formula-based fortifiers to support gut function, nutrient absorption, and bacterial defense in preterm pigs [61], the oral administration of colostrum does not seem to reduce NEC onset from recent meta-analyses [62].

Regarding initiation and advancement of enteral feeds, recent systematic reviews demonstrated that trophic feeds can be started within 96 h from birth and at higher volumes without affecting the risk of NEC in VLBWI [63,64]. However, limited data exist for infants born <28 GA or below 1000 g. A slower advancement of enteral nutrition (18 mL/kg/day) does not seem to lower the risk of NEC compared to a faster one (30 mL/kg/day) [65,66]. In addition, there appears to be no difference in the incidence of NEC when infants <37 GA and with a BW < 2500 g receive bolus feeding compared to continuous ones [67].

Considering routine monitoring of stomach aspirates, up to February 2018 there were no adequate data to support this strategy as a guide to initiate and increase feeds in healthy preterm infants. This practice, if not uniformly standardized, may lead to delay in reaching full feeds, longer duration of PN and central line usage and, as a consequence, to more potential complications such as late-onset sepsis [68,69]. The implementation of standardized feeding protocols to manage trophic feeds, residuals, and timing of advancement of feeds may help in lowering NEC rates [70].

#### 2.2.2. Drugs and Anemia

Among other early interventions that can be adopted, although with still low to moderate evidence so far, there is the avoidance of prolonged antibiotic therapy, hyperosmolar agents, histamine 2 blockers and severe anemia.

Disruption of commensal bacterial colonization with overgrowth of potentially pathogenic bacteria plays an important role in NEC development. Findings from observational studies show that early antibiotic administration or exposure greater than 5 days can be associated with an increased risk of NEC [71,72,73].

Hyperosmolality has been historically thought to increase the risk of NEC, but animal and human studies have reported contrasting results [74]. Hyperosmolar medications, such as multivitamins, contrast agents, and hypertonic additives of certain oral drugs could cause mucosal injury and therefore increase the risk of NEC [75]. Similarly, agents that reduce gastric acidity, such as histamine type 2 (H2) receptor antagonists, can lower the inhibitory effect of gastric pH on bacterial growth [76], as illustrated by a report from the National Institute of Child Health and Human Development Neonatal Research Network on more than 11,000 preterm infants, where those treated with H2 antagonists had higher odds of NEC compared to controls [77].

Despite ischemic insults to the gastrointestinal tract have long been proposed to be a contributor to NEC, there are still inadequate observations to confirm it. Previous studies had hypothesized a role of red blood cells (RBC) transfusion 48 h prior to the development of NEC, but this has been confuted by results of a large multicenter cohort study on VLBWI [78]. This study revealed that rather than RBC transfusions, is severe anemia (hemoglobin level ≤8 g/dL) within the week of developing NEC that could predispose to the disease. The association between NEC and RBC transfusion has been discredited even by a recent review of the literature [79]. Furthermore, in a Cochrane review published in 2019 there were insufficient data to evaluate whether stopping feeds during blood transfusion is helpful in preventing the disease [80].

#### 2.2.3. Immunologic Stimuli

In recent years, there has been a growing interest for immunomodulatory agents, such as probiotics, lactoferrin, immunoglobulins and nutritional supplements, which however have not proven to be effective in reducing the burden of NEC.

Compared to placebo, probiotics seemed to provide positive results in several small sample-sized studies [81,82]. Nevertheless, the optimal strain, dosing and timing of their administration still need to be established [83], and concerns arouse in the past due to the reported cases of sepsis related to their administration [84]. Recently a position paper has been released by the ESPGHAN Committee on Nutrition and the ESPGHAN Working Group for Probiotics and Prebiotics on the probiotic strains with greatest efficacy regarding relevant clinical outcomes for preterm neonates. This paper favors the use of Lactobacillus rhamnosus GG ATCC 53,103 or of a combination of Bacillus infantis Bb-02, Bacillus lactis Bb-12 and Streptococcus thermophilus TH-4 to reduce NEC Bell’s stage II and III, although with low certainty of evidence [85]. As concluded by a recent Cochrane review of the literature, further, large, high-quality trials are needed to provide evidence of sufficient quality and applicability to inform policy and practice [86].

Similarly, no recommendation can be made regarding the use of oral immunoglobulin (IgG alone or IgG plus IgA) [87] nor of enteral lactoferrin as an adjunct to antibiotic therapy for the prevention or treatment of NEC [88]. In fact, the former did not reduce the incidence of definite NEC, suspected NEC, need for surgery, or death from NEC in a meta-analysis of five RCTs, while the latter did not improve the rates of Bell stage II and III (proven and advanced) NEC nor that of late-onset sepsis compared to placebo in the largest RCT [89].

**Figure 1:**
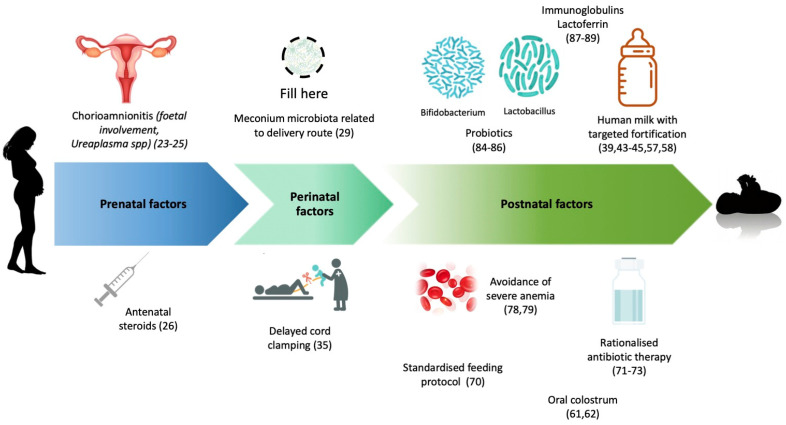
Prenatal, perinatal and postnatal factors which have been found, with stronger or weaker recommendation, to reduce the subsequent risk of NEC development. The role of certain factors, like chorioamnionitis, meconium at birth, delayed cord clamping, oral colostrum, is still debated. Icons from Freepik and Pch.vector, downloaded from freepik.com.

A summary of antenatal and postnatal factors involved in NEC pathogenesis is reported in Figure 1.

## 3. Improving NEC Diagnosis: Indicators of Suspected NEC

Given the frequently sudden onset and the potential devastating effects, it is of extreme importance to apply a combination of imaging and monitoring tools to early recognise patients at risk and act before development of the disease. Studies conducted so far have predominantly explored laboratory features and thresholds that could reveal the incipient onset of NEC, and their results have been extendedly described elsewhere [90,91].

Relatively new interesting approaches, such as metabolomic and microbiota analysis, have been applied on serum, urine and fecal samples to investigate prognostic factors of NEC onset. Studies using these techniques have been mostly prospective and have included small sample sizes of matched NEC infants and controls. Some of them have revealed that a NEC-associated gut microbiota can be identified in meconium or pre-NEC stool samples [92], with an increased relative abundance of Proteobacteria and Firmicutes, and decreased relative abundances of Bacteroidetes prior to NEC onset [93]. Among the Preoteobacteria, Escherichia coli and Klebsiella pneumoniae seem the most pathogenic, while Clostridia are the most abundant among Firmicutes. Bifidobacteria, instead, are often lacking in pre-NEC stools of affected patients [94].

Non-invasive parameters have been advocated to continually monitor premature neonates in order to early detect predictive changes. The monitoring of transcutaneous PO_2_ (tcPO_2_) has proven to be safe and accurate in very sick infants [95,96] with a good linear correlation between tcPO_2_ and Partial Pressure of Oxygen (PaO_2_) [97]. In one study, drops of the tcPO_2_/PaO_2_ ratio could be the spy of NEC requiring surgical intervention, with appropriate response to fluid resuscitation in survivors [98].

Near Infrared Spectroscopy (NIRS) assessment of neonatal splanchnic oxygenation (SrSO_2_) has gained increasing interest over the last decade. The infraumbilical abdomen is considered the most reliable area for sensor placement [99]. In preterm neonates, a reliable correlation between SrSO_2_ and mesenteric Doppler has been reported [100], with evidence supporting the feasibility of NIRS in the monitoring of enteral feeding [101] and NEC development [102,103]. Lower infraumbilical SrSO_2_ and higher Fractional Oxygen Extraction within twenty-four hours after onset of symptoms suspicious of NEC can predict subsequent gastrointestinal complication (Bell’s stage IIIB or death) [103]. Interestingly, Doppler and NIRS techniques have been combined in the study of transfusion-associated NEC and confirmed a possible pathogenic role of the pre-existing severe anemia rather than of RBC transfusion in the onset of NEC [104]. The same authors found that feeding during RBC transfusion, instead, was related to a post-prandial decline in the postprandial mesenteric oxygenation as measured by SrSO_2_ [105]. The splanchnic–cerebral oxygenation ratio has been proposed as an index to predict splanchnic ischemia [106].

Abdominal ultrasound has become more and more popular in the diagnostics of necrotizing enterocolitis thanks to its non-invasiveness, quick use, and good performance also in equivocal cases at abdominal radiography. With good accuracy typical signs of NEC can be recognized, such as intestinal wall thickness and pneumatosis (hyperecogenic foci), intestinal loops’ peristalsis and dilation, ascites, pneumobilia and pneumoperitoneum. Color Doppler may reveal perfusion of the intestinal wall and flows in the abdominal aorta and mesenteric vessels, with recognition of subtle hyperaemia of bowel loops or lack of flow [107]. Interestingly, a recent single-center study in 104 preterm neonates showed a promising value of Doppler ultrasound of the superior mesenteric artery as additional prediction surrogate to predict NEC. In particular, a higher peak systolic velocity and differential velocity measured in the superior mesenteric artery within the first 12 h of life were significantly related to the risk of NEC [108].

Further studies are needed on the use of laboratory values, perfusion indices and imaging techniques for the early recognition of NEC.

## 4. Surgical Treatment: Control of Long-Term Consequences

### 4.1. Surgery Aims

For Patients with NEC refractory to maximal medical treatment (multi-organ failure, progressive clinical deterioration) or with perforated NEC, surgery is indicated. In the current literature, the percentage of patients requiring surgical treatment is consistently between 20% and 50% [109,110].

Several different operative management are described which vary between teams from minimalistic strategies (e.g., peritoneal drainage) to demolitive laparotomy.

The surgery leading principles are:

− to prevent the short bowel syndrome (limit the resection)
− to limit bacterial translocation by diverting feces
− to reduce the risk of sepsis and control the inflammatory cascade (by resecting necrotic bowel), reducing the consequent risk of multi-organ failure.

### 4.2. Surgery Options

Deciding the type of operation to perform depends on the extension of the disease but also on the surgeons’ experience.

#### 4.2.1. Stoma Versus Primary Anastomosis

The most traditional surgical approach for NEC is to perform a laparotomy with diverting stoma proximal to most diseased bowel. This procedure has some advantages, mainly of allowing the heal of downstream bowel without translocation of stool/bacteria. Some centers consider refeeding the proximal stoma effluent through the distal mucous fistula in order to stimulate mucosal growth and minimize fluid and electrolyte losses. However, there is little evidence to support the efficacy of this practice [111,112]. Stomal complications including fluid losses, electrolyte abnormalities, poor growth, prolapse or retraction of stoma led some groups to consider primary anastomosis as the first option to treat severe NEC. Guelfand et al. [113] reported primary anastomosis as a safe procedure in the treatment of complicated NEC with low morbidity and mortality (11.6%). A recent systematic review [114] of 12 studies compared these two approaches and found no significant difference in terms of complications or mortality rate, although the spectrum of complications was slightly different in each group, potentially due to selection bias for treatment options and heterogeneity of included studies. Similarly, a survey of the European Pediatric Surgeons’ Association (EUPSA) reported that the majority of surgeons (67%) opted for bowel resection and primary anastomosis in the case of focal NEC, while 75% would perform a stoma in case of multi-focal disease [115].

#### 4.2.2. Peritoneal Drainage

In VLBWI, a minimalistic strategy is often advocated, consisting of in the placement of a peritoneal drainage (PD) without intestinal exploration in case of free air at the X-ray. This procedure was firstly described by Ein et al. in 1977 [116] as a way to stabilize neonates until they are in better conditions to undergo an explorative laparotomy. Over time, PD has become popular and some pediatric surgeons consider PD not only as a temporizing measure but also as a definitive treatment. Tashiro et al. showed that in premature (<37 GA) and extremely low birth weight infants (BW < 1000 g) with severe NEC, PD was associated with a higher survival rate compared to primary laparotomy, either considering PD as a definitive treatment or as a bridge before explorative laparotomy [117]. As the best of our knowledge, only two randomized trials that compared PD with laparotomy were conducted: the North American trial, published in 2006 (NECSTEPS trial) [118] and the European Trial in 2008 (NET Trial) [119]. Moss et al. considered VLBWI with intestinal perforation and found no significant differences between the two groups in the primary outcomes of 90-day mortality, 90-day dependence on total parenteral nutrition (TPN) or length of hospital stay [118]. Similarly, Rees et al. considered extremely low birth weight infants (ELBWI) with BW < 1000 g with pneumoperitoneum on radiography and found no difference in 1-month mortality or 6-month mortality [119]. A Cochrane meta-analysis of both randomized control trials did not show differences between the two groups in terms of survival rate, as well [120].

**Figure 2:**
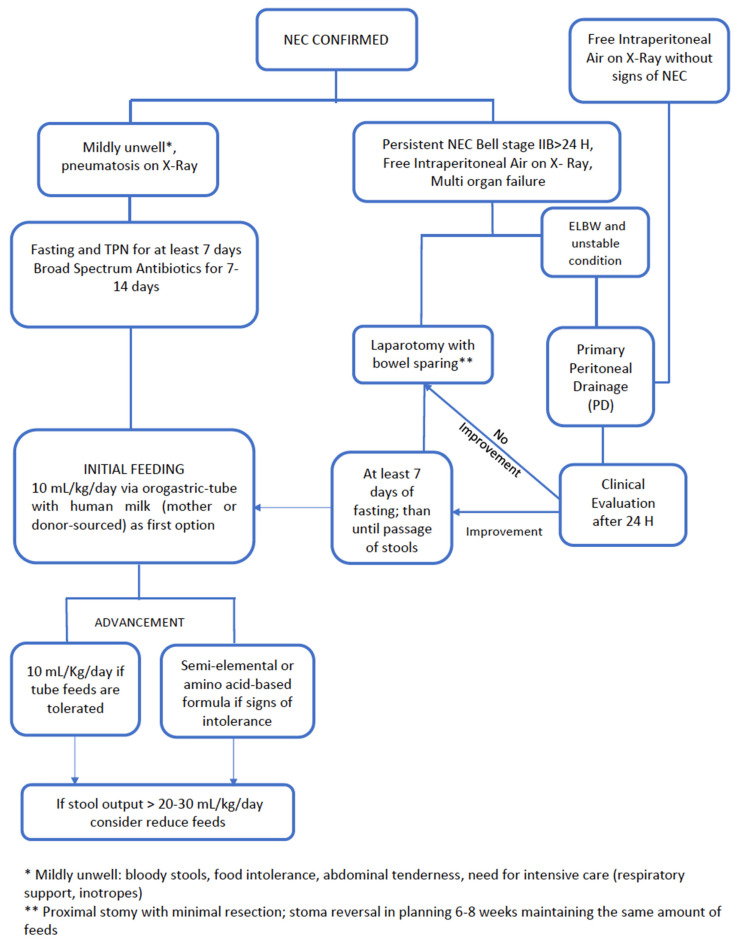
Our center Policy for infants with NEC.

Our institution policy consists of performing surgery in case of persistent NEC Bell stage IIB for more than 24 h, free intraperitoneal air detected on radiological examination, and worsening of multi organ failure. When laparotomy is performed, minimal bowel handling is recommended avoiding resection. PD is advocated in ELBWI with unstable conditions (inotrope need, maximal ventilator support) when perforation is suspected or when free intraperitoneal air is detected on X-ray without previous clinical or radiographic signs of NEC. The latter condition is highly suspicious for isolated bowel perforation. Absence of improved condition and enteral output from PD after 24 h are indications for delayed laparotomy (Figure 2). As previously described, this policy based on the concept of “sparing surgery” is safe and it seems to be associated with a lower mortality rate than the one reported in the literature (6.4%) [121].

### 4.3. NEC Totalis Management

A clinician’s challenge in the management of NEC is the NEC totalis (NEC-T), where the risk of SBS is very high. No univocal definition is reported in the current literature, as according to some groups NEC-T occurs when the entire small intestine is involved, whereas for others when the necrosis involves the small and the large bowel [122]. In addition, defining the bowel vitality in a pan-intestinal necrosis is not always straightforward and it may result in unnecessary extensive resection. Furthermore, defining the real extension of the disease requires the surgical exposition of the whole bowel, with the aforementioned danger. In 2004, Pierro et al. proposed the use of gasless laparoscopy to define the length of intestine involved by necrosis [123]. Many different surgical approaches have been described. In 1989, Moore et al. introduced the “patch, drain and wait technique” [124]. This approach consists of covering the perforation with sutures (patch), positioning two drains (drain) and then waiting with long-term parenteral nutrition (wait). The critical aspects of this type of treatment is the uncontrolled clearance of the abdomen from necrotic tissue and feces. In 1996, Vaughan et al. proposed the “clip and drop technique” that also aims to avoid ostomies and to preserve intestine length [125]. This technique involves resection of necrotic bowel and tiding off the ends of healthy bowel tracts. A second-look laparotomy after 48–72 h is used to determine the true extent of the disease and intestinal continuity is restored. Arnold et al. reported their experience using this strategy and found lower incidence of SBS (9.1%) compared to 16% in the published data [126]. Therefore, they suggested the “clip and drop” technique in selected patients with NEC-T to help bowel conservation in survivors.

Alternatively, intraluminal stenting could be used to preserve the length of the intestine as much as possible in these sick infants [127]. The management of NEC-T remains very controversial and significant practice variability persists. Nevertheless, it raises ethical issues. In fact, the total-length extension of necrosis gives scarce chances of bowel function recovery. In the pre-parenteral nutrition era, palliation was the choice rather than surgical techniques to keep the patient alive. Presently, in developed countries tailored PN and later in life the bowel transplant option, impose to consider and treat these patients. If no healthy small bowel can be found at the laparotomy, tube duodenostomy can be used as a temporizing maneuver in these neonates.

### 4.4. Complications

Intestinal strictures after NEC, firstly described by Rabinowitz 1968 [128] is a well-known common complication of NEC, affecting about 9–36% of patients [129,130]. Differently from the onset of NEC which is most frequent in the terminal ileum/cecum, the most common site of post-NEC-stricture is the left colon (80%). In the current literature, some studies focused on predictive factors of this complication in order to avoid intestinal resection. A retrospective study by Phad et al. [131] found that leucocytosis during NEC and length of resected bowel at surgery may be associated with increased risk of developing post-NEC intestinal stricture. In their multicenter study, Zhang et al. showed that the late onset of NEC (>10 days of life) and the higher level of procalcitonin at the onset of NEC could be consider independent factors for post-NEC strictures [132]. A recent meta-analysis showed that earlier feeding <7 days after a NEC diagnosis should be considered to be a way to reduce the risk of stenosis. However, this review did not include randomized trials and patients who started feeding earlier could have been less sick [133]. Considering that the classical approach for severe NEC has been to fashion a stoma, metabolic disturbances and poor growth are more frequent in these patients as a consequence of electrolyte depletion. To avoid these complications, it has been proposed to reestablish bowel continuity as soon as possible [134,135,136]. This would theoretically restore the bowel transit and thus prevent the strictures of unused tracts. However, most common practice is to wait for at least 6–8 weeks after the first surgery, when patients have greater body weights and the inflammatory changes are settled [137]. Our policy is to reverse the stoma in 6 weeks (if the management of ostomy’s losses allows it), and when the patient is growing properly.

A recent systematic review demonstrated that there were no significant postoperative complications after stoma reversal between early and late stoma closure [138]. However, this review mainly included retrospective studies, with differences in the management of NEC and small number of patients. Again, a RCT should be planned to demonstrate the superiority of late versus early closure of stoma. In addition, further prospective trials are needed to evaluate the outcomes of existing approaches.

## 5. Best Nutrition Strategies to Enhance Intestinal Adaptation and Sustain Growth

The origin, development, and treatment of NEC are still being debated. Nonetheless, while certain feeding practices are recognized preventive factors, there is no consensus on when and how reintroduce enteral feeding after NEC and in SBS patients after surgery.

### 5.1. When to Start Nutrition

After NEC diagnosis, bowel rest is suggested for 7–10 days [139,140]. However, the reason for a long period of fasting is unclear since the presence of macro and micronutrients in the intestinal lumen enhance intestinal adaptation [141,142,143].

Since feeding, together with prematurity and altered gut colonization, is one of the key factors that triggers the inflammatory cascade leading to NEC, clinicians are afraid of restarting enteral nutrition because of the possibility of recurrence of NEC. Recurrent NEC was reported with an incidence of 4–6% [144,145], with a higher incidence in those refed before 10 days after an episode of NEC [146]. However, other studies reported no complications associated with an earlier refeeding practice after NEC [147,148,149].

In a retrospective study, Bonhorst et al. [148] compared patients subjected to a new protocol of feeding practice with an historical group. The more recent group of newborns was fed with a median of four days after three consecutive days without evidence of gas bubbles in the portal vein studied with the use of abdominal ultrasound. This group was compared with a cohort group fed on the basis of the neonatologist discretion (median 10 days). The early feeding group reached full enteral feedings in significantly less days (10 days vs. 19 days), had a reduced duration of central venous access (13.5 days vs. 26.0 days) and had a lower rate of catheter-related septicemia (18% vs. 29%,) with consequent shorter length of hospital stay (63 days vs. 69 days).

Other studies, though considering only non-surgical NEC, retrospectively analyzed the outcomes of patients receiving early (<5–7 days) or late feeding (>5 or ≥7 days) [147,149]. Brotschi et al. [149] found that neonates refed in less than 5 days developed less catheter-related sepsis and required less surgery for early post-NEC stricture. Arbra et al. [147] retrospectively reviewed the ten-years data in a single center and analyzed outcomes in patients fed at < vs. ≥7 days from NEC diagnosis. After adjusting for NEC stage, the composite outcome for stricture, recurrence of NEC or death was not significantly different between the early and late refeeding groups.

Two Metanalyses have been performed on refeeding practices after NEC [133,150]. These found that early enteral feeding (within 5 days of NEC diagnosis) did not seem to be associated with adverse outcomes, including NEC recurrence.

Early enteral feeding is important to prevent gut atrophy and to improve intestinal growth in parenterally fed preterm newborns [151]. In a rat model of NEC, a 25% reduction of enteral nutrients resulted in a reduced villus height and gut mass [152] while early feeding after surgery was associated with improved intestinal adaptation in piglets [153].

Given this evidence, several experts report that enteral feeding should start as soon as possible in newborns after surgery to stimulate gut adaptation [7,154,155,156], though no clear guidelines have been established yet [115]. Other experts, in contrast, state that enteral feeding should start when bowel sounds are present, enteral drainage is no longer bilious, the abdomen is soft and there is no vomiting [157].

### 5.2. Type of Enteral Feeding

To our knowledge, there is no trial aimed at verifying the best type of feeding after NEC resection. There is a large consensus reporting human milk as best choice of feeding for newborns and infants with SBS [44,156,158,159,160,161]. Breast milk is rich in proteins (immunoglobulin A), nucleotides, live cells, and growth factors together with other components such as lactoferrin and more than 100 different oligosaccarydes that can reinforce the immune system and enhance intestinal growth and adaptation [162,163,164,165].

Human milk is recommended by the Enhanced Recovery After Surgery Society as first choice of nutrition in the newborn after surgery [166]. In the absence of human milk, it is still debated the best type of formula milk to use. Several NICUs use extensively hydrolyzed protein formula in the absence of human milk [167] as recommended by some experts [168]. The use of Extensively Hydrolyzed Formula (EHF) is justified by the increased risk of protein allergy characterizing newborns after intestinal surgery [169,170] and by the possible relationship between protein’s allergy and NEC [171,172]. EHFs do not contain lactose and usually have an increased concentration of medium-chain triglycerides (MCT). It seems therefore to be preferable if it is taken into account that undigested lactose is a contributing factor to NEC development in animals [173] and that preterm newborns have a reduced lactase activity compared to their term counterparts [174]. In addition, components such as MCT can be better absorbed in the case of rapid transit, bacterial overgrowth, and bile acid depletion [175] as it is after surgical NEC. Nonetheless, the possible disadvantages of EHF should be considered, since it does not satisfy the elevated requirements in preterm infants [176]. As far as we know, the only randomized study comparing hydrolyzed vs. non-hydrolyzed formula in children with SBS did not detect differences in tolerance and weight gain [177]. Furthermore, breast milk together with elemental formula (aminoacid-based) resulted in shorter parenteral nutrition dependence compared to the hydrolyzed formula [20,178,179,180,181,182].

Finally, macronutrients in a complex form were found to better promote bowel adaptation in animal models [183,184,185,186], outlining the importance of bowel workload in enhancing adaptation [187].

For the aforementioned reasons, some authors suggested to feed premature infants with preterm formula, in the absence of human milk, and then verify its tolerance [187].

More recently, the use of cow’s preterm formula has been recommended in the absence of human milk [158], since it is characterized by higher caloric density and a composition based on lower lactose, higher MCT and long-chain triglycerides contents with the well-known advantages of these lipids [188]. The use of semi-elemental or elemental formula is suggested in those patients who are intolerant to conventional preterm/term formula [159,160].

### 5.3. How to Increase Feeds

After an episode of NEC requiring surgery, most of the studies suggest starting feeds with 10 mL/kg/day initially [148,149,157] and then advance by increasing from 10 to 20 mL/kg/day [188] or 20 mL/kg/day [148,149]. Others, however, suggest a more cautious approach with smaller advancements (1–2 mL every 3 h for 24–48 h or 0.5–1 mL/kg/day) [141,143]. Especially in ELBWI who were never previously fed, some authors report to increase 1 mL every 4 h for 5 days [189].

There is a quite uniform consensus to limit advancing of feeds when stool/stoma output is more than 30–50 mL/kg/day [7,157,188,189,190], or when it is more than 20 mL/kg/day or with a stool production of >6–10 times/day [157,191]. It is, therefore, mandatory for the clinician to carefully quantify stool number/volume [157] and to observe possible clinical changes (vomit, bowel distention, irritability) [168] before increasing enteral nutrition.

There is no clear preference in the literature for the feeding method (continuous vs. bolus) in preterm [192] infants after bowel resection [193]. Despite of the fact that bolus feeding increases splanchnic perfusion with an improvement in digestion [194], growth of VLBWI could benefit from continuous feeding [195]. Continuous feeding is suggested in newborns and infants with SBS [7,155,158,160] at the beginning of the refeeding process, at least during nighttime [196] or for the first 24 h [160]. It is reported that continuous feeding may enhance absorption and improve growth in selected groups of patients [197].

On the other hand, both animal [198] and human studies [199] demonstrate that bolus feeding is more physiological, increases mucosal mass and enzyme content and bowel adaptation [190].

A practical approach could be to start with continuous feeding followed by bolus feeding, and as soon as possible introduce small volume of feeding orally administered [159,193] to stimulate swallow reflexes and avoid later full aversion disease [200,201].

In general, the evidence shows that the use of a standardized refeeding protocol results in fewer days to achieve the 50% of enteral nutrition and in less Intestinal Failure Associated Liver Disease (67% vs. 42%) [202].

### 5.4. Optimizing Parenteral Nutrition and Laboratory Controls during Refeeding

During the refeeding process, it is fundamental to maintain the potential growth of the newborn, despite SBS avoiding both over- and underfeeding. The new 21st Century Preterm Postnatal Growth Standards Charts from birth to 6 months of corrected age can help the clinician to monitor growth and provide the right macro and micronutrient requirements after bowel resection [203].

Although energy expenditure seems to be unaltered in surgical neonates [204], there is a paucity of data in preterm infants. In addition, these subjects face a very rapid cerebral and organ growth which requires high nutrient and caloric intakes compared to their term counterparts [205,206].

Macro and micronutrients must be guaranteed through the parenteral route when enteral nutrition is insufficient to meet required intakes. PN is important in all the phases after bowel resection: In the first phase soon after bowel surgery, when the newborn is completely dependent and aggressive fluid and electrolytes replacement are warranted; in the second phase, when enteral nutrition is started; in the third phase when all the efforts are directed toward PN weaning [158]. The necessity of maintaining growth has to be balanced with the excess of macronutrient intakes by PN.

The excess of w6 Long Chain Tryglycerides, for instance, can adversely affect the liver due to the pro-inflammatory and pro-oxidative actions [207]. Soy emulsion are rich in phytosterols that by the enteral route would be only poorly absorbed, but by the parenteral route may lead to liver damage. In preterm infants, especially in those with cholestasis, phytosterols have longer half-life [208,209,210], therefore exposing these subjects to an increased risk of liver injury. For this reason, recent guidelines on lipid intakes report that for “PN lasting longer than a few days, pure soybean oil based intravenous lipid emulsions should no longer be used, and composite Intravenous Lipid Emulsions with or without fish oil should be the first choice” [211].

Higher dextrose and amminoacids intakes, as well, may cause increased prevalence and earlier onset of PN -Related Cholestasis [212]. On the other hand, it should be kept in mind that some non-essential amminoacids, such as taurine and cysteine, may become conditionally essential in preterm newborns, who therefore could benefit from their supplementation [213,214] together with the fact that in some cases an amminoacids’ amount up to 3.5 g/kg/day should be administered to prevent catabolism [215].

It is, therefore, recommended to adjust energy intakes on patient’s condition and to avoid excess energy intakes by PN maintaining a nonprotein carbohydrates/lipids ratio of 75/25 with an upper triglyceride level in the newborn of 2.5 g/L during lipid infusion [201].

Preterm infants with SBS need to receive macronutrient but also micronutrients (such as iron, zinc, copper, selenium, Vitamins) and electrolytes (calcium magnesium, sodium) to prevent their deficiencies [216,217,218,219].

For this purpose, a strict monitoring of laboratory values reflecting liver function, nitrogen content, renal function, and electrolytes and vitamin levels is of extreme importance for infants [220] and newborns on long-term PN [155] keeping in mind that electrolytes serum levels could seem appropriate despite a low total body content. Levels of electrolytes in urine (potassium, sodium, magnesium), should be routinely performed as well. In particular, urinary sodium should be kept >20–30 mEq/L [220,221] to meet the elevated newborn’s requirements for growth.

To conclude, nutrient intakes must be adapted according to the newborn’s nutritional requirements and through frequent anthropometric and biochemical assessments [155,156].

Table 1 describes the feeding protocols adopted to initiate or advance after surgery for NEC.

## 6. Conclusions

Necrotizing enterocolitis is still an emerging disease in preterm newborn infants carrying a high morbidity and mortality rate. There are several factors that appear to be promising in preventing its onset, such as antenatal steroids, human maternal or donor milk, and targeted fortification of feeds. At the same time, prediction tools such as abdominal NIRS or abdominal ultrasound should be implemented to detect patients at risk early. After diagnosis, it is fundamental to customize the medical and surgical management in order to limit and treat NEC complications, especially short bowel syndrome. An efficacious and customized parenteral nutrition and early refeeding with human milk play a key role in these patients. An individualized follow-up based on growth and focused on avoiding nutrients deficiencies is mandatory. Nutritional strategies with standardized protocols for refeeding after surgery play a key role in this sense.

## Figures and Tables

**Table 1:** Summarizes the Proposed Refeeding protocols after NEC in the current literature.

Authors and Journal	Year	Initial Feeding	Advancement	Type of Feeding	Type of Feeding in Absence of Human Milk
**Christian V.J. et al. (Nutrition in Clinical Practice) [158]**	2018	Continuous feeds: 20 mL/Kg/day	10–20 mL/kg/day	Human milk (Mother or donor)	Preterm/term formula - If patient is intolerant: semi elemental or amino acid-based formula
**Shores D.R. et al. (Journal of Perinatology) [189]**	2015	Bolus: 20 mL/Kg/day or 15 mL/kg/day in VLBWI	15–20 mL every 12–24 hours	Human milk (Mother or donor)	Elemental Formula
**Brotschi B. et al. (Journal of Perinatal Medicine ) [149]**	2009	Bolus: 10 mL/kg/day	20 mL/Kg/day to 140–150 mL/kg/day	Human Milk (Mother or donor)	Formula milk
**Parks P. et al. (Practical Gastroenterology) [160]**	2008	/	Continuous feeds: 10–35 mL/kg/day	Human Milk (Mother or donor)	Preterm/term formula - If severe NEC: Semi elemental or amino acid-based formula
**Bohnhorst B. et al. (Journal Pediatric) [148]**	2003	20 mL/kg/day	20 mL/kg/day to 150 mL/Kg/day	Distilled water followed by Human Milk (Mother or donor)	Distilled water followed by Full-strength formula
